# Tuberculosis Presenting as a Patchy Consolidation in a Young Patient With Chronic Granulomatosis Disease

**DOI:** 10.1002/ccr3.70279

**Published:** 2025-02-25

**Authors:** Davood Attaran, Shima Baniassad, Zahra Behrooznia, Ehsan Taheri, Soroush Attaran, Amir Baniasad

**Affiliations:** ^1^ Lung Disease Research Center Mashhad University of Medical Sciences Mashhad Iran; ^2^ Department of Anesthesiology, Sina Hospital Medical Sciences University of Tehran Tehran Iran

**Keywords:** chronic granulomatous disease, *Mycobacterium tuberculosis*, opportunistic infections, pneumonia

## Abstract

A 27‐year‐old male with known CGD presented with severe dyspnea and a productive cough. Following clinical and diagnostic evaluations, he was diagnosed with pulmonary tuberculosis. This case highlights the challenges in diagnosing TB in patients with CGD and the need for more precise diagnostic approaches when treating this vulnerable population.

## Graphical abstract

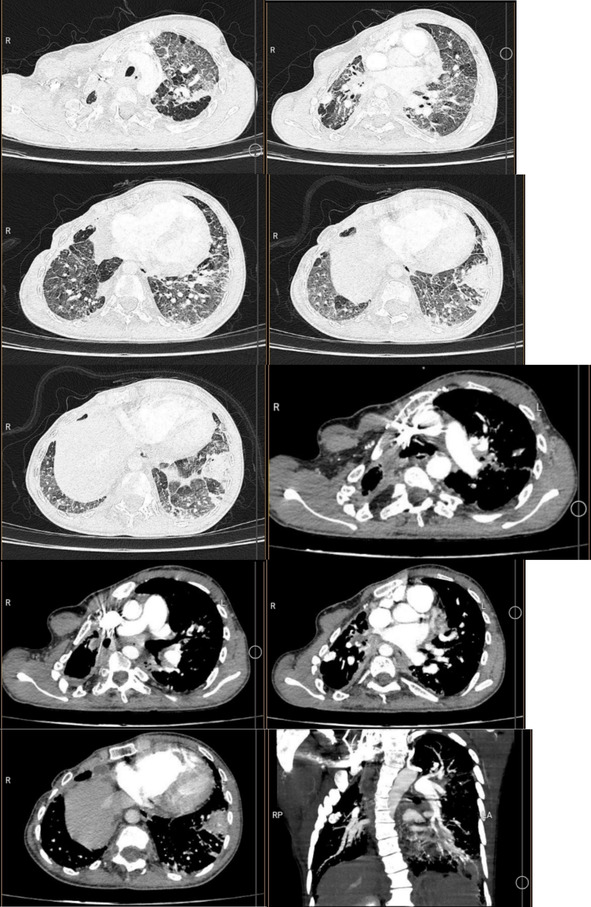

## 1 Introduction

Chronic Granulomatous Disease (CGD) is a rare disease caused by the dysfunction of phagocytes caused by defective nicotinamide adenine dinucleotide phosphate (NADPH) oxidase. These patients suffer from frequent bacterial and fungal infections, and tissue granuloma formation is one of the characteristics of this disease [1]. The most commonly involved organs in this disease are the lungs, lymph nodes, liver, and skin. CGD is usually diagnosed between the ages of 1 and 3 years. Milder forms of the disease appear older and have a longer lifespan [2].

Known treatments such as prophylactic antibiotics, antifungals, and interferon (IFN)‐γ have improved the outcome, but infections are still associated with high morbidity and mortality in these patients. The lung is the most common site of infection in CGD patients, and 87% of patients experience at least one episode of lung infection in their lifetime [3].

The almost high rate of infections in CGD patients despite prophylactic treatments can be due to inescapable exposures to environmental infections and the complexity of prophylactic treatment in a disease with an intermittently reinforced nature [4].

Infection with *Mycobacterium tuberculosis* (the causative agent of tuberculosis (TB)) in immunocompromised patients such as CGD can cause atypical and more severe manifestations that complicate the diagnosis and treatment of these patients [5]. Signs and symptoms may include fever, fatigue, malaise, chronic cough, dyspnea, leukocytosis, or neutrophilia; however, these symptoms can be mistaken for other infections or complications related to CGD [5].

Considering possible atypical manifestations of TB in CGD patients and its diagnostic challenge, we report a 27‐year‐old male patient with a known case of CGD who presented with shortness of breath and a productive cough. Following clinical and diagnostic evaluations, he was diagnosed with pulmonary tuberculosis.

## 2 Case History/Examination

A 27‐year‐old male patient with a known case of CGD was admitted to the emergency room with the chief complaint of severe dyspnea and a productive cough. The dyspnea and productive cough began 20 days ago. The patient's dyspnea was progressive and in such a way that the patient had shortness of breath during routine daily activities. The patient already had occasional non‐productive coughs, which increased and became productive 20 days before the patient's visit. The patient has had occasional fevers for the past 3 weeks. He did not have recent weight loss.

The patient had a known case of CGD since 5 years old, with the initial manifestation of recurrent Pneumonia. At the same 5 years old, he had undergone lobectomy of the right lower lobe due to localized bronchiectasis. The patient had occasional generalized tonic‐clonic seizures 5 years ago, and treatment with antiseizure medications was administered for the patient. One year ago, the patient had a pulmonary embolism (PE), which was treated with apixaban (5 mg twice daily) for 6 months. He has been hospitalized many times due to Pneumonia; the last one was 1 year ago. The patient was diagnosed with Aspergillus pneumonia in the last hospitalization (1 year ago).

The patient has been treated with co‐trimoxazole (400/80 mg twice daily), levofloxacin (500 mg once daily), itraconazole (100 mg twice daily), levetiracetam (500 mg twice daily), carbamazepine (200 mg twice daily), sodium valproate (500 mg twice daily), escitalopram (20 mg once daily), N‐acetylcysteine (600 mg once daily), tiotropium (18 mcg/capsule inhaled once daily), and Salmeterol/fluticasone propionate (125/25 μg) (one inhalation twice daily).

The patient had no history of smoking or opium addiction. The patient had no history of similar pulmonary disease in first‐ and second‐degree relatives.

In the clinical examination at the emergency room, blood pressure was 95/63 mmHg, respiratory rate was 33/min, pulse rate was 140/min, temperature was 37.8°C, and oxygen saturation was 78% without supplemental oxygen and 96% with a nonrebreathing oxygen mask. The patient's weight and height were 51 kg and 173 m, respectively.

The patient was not pale in the clinical examination. There was no cyanosis in the examination of the lips and oral mucosa. He had fine crackles in the bases of both lungs. There was no edema or difference in size in the examination of the extremities.

## 3 Methods

**FIGURE 1:**
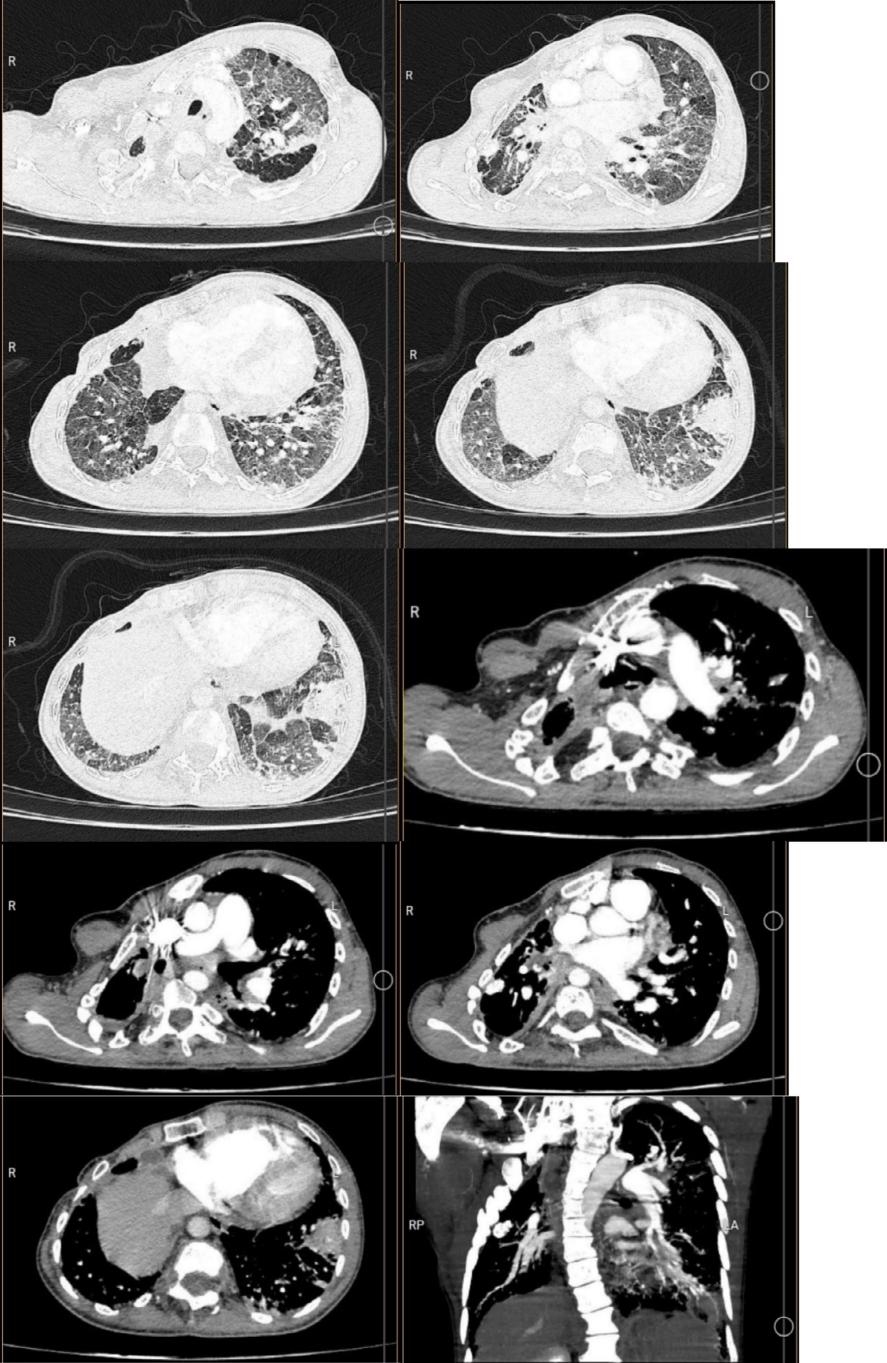
Primary lung CT angiography of the patients. There was no evidence of pulmonary embolism. There was centrilobular and paraseptal emphysema in the lung parenchyma, with a preference for the upper lobes. Scattered ground glass opacities were observed. There was a consolidation in the left lower lobe. A 20 mm calcified nodule in the right upper lobe was observed.

Based on the clinical history and examination, our differential diagnosis was infection due to bacterial, viral, and opportunistic infections. Another differential diagnosis was PE. The patient's coronavirus disease‐2019 (COVID‐19) polymerase chain reaction (PCR) test was negative. Considering the previous history of PE and the sudden onset of shortness of breath and tachycardia, lung CT angiography was performed for the patient with suspicion of PE. No new embolism was seen in the imaging, and the only evidence of centrilobular and paraseptal emphysema was observed in the lung parenchyma, with a preference for the upper lobes. Also, scattered ground glass opacities and consolidations in the left lower lobe were observed (Figure 1).

The patient was transferred to the ICU, and due to respiratory distress, noninvasive ventilation (NIV) by Bilevel positive airway pressure (BIPAP) was started. Considering the patient's past medical history of CGD and patient's clinical and radiologic findings, including hypoxemia, empiric antibiotics for coverage of gram‐negative pathogens (like pseudomonas), besides routine pathogens, were essential, so the patient was treated with ciprofloxacin (400 mg twice daily), meropenem (1 g every 12 h), and methylprednisolone (40 mg once daily). Other drugs that the patient was already using were also prescribed by the prior dosage to the patient (co‐trimoxazole, itraconazole, levetiracetam, carbamazepine, sodium valproate, escitalopram, N‐acetylcysteine).

In the patient's echocardiography, he had an ejection fraction of 45%, right ventricular enlargement, severe tricuspid regurgitation, systolic pulmonary artery pressure (sPAP) of 65 mmHg, mild to moderate mitral regurgitation, and global hypokinesia. A diuretic (furosemide 20 mg twice daily) was prescribed to the patient.

During hospitalization, hypoxia and the patient's general condition improved relatively. However, 11 days after the patient's admission, he developed a fever again, and vancomycin (1 g every 12 h) was added to the patient's antibiotic treatment.

The patient's fever continued despite treatment with three intravenous antibiotics, so a CT scan of the sinuses and lungs, sepsis workup, and examination of sputum for Pneumocystis, Mucormycosis, and Aspergillus PCR were performed for the patient. The bronchoscopy was not performed for the patient due to pulmonary hypertension and hypoxia.

**FIGURE 2:**
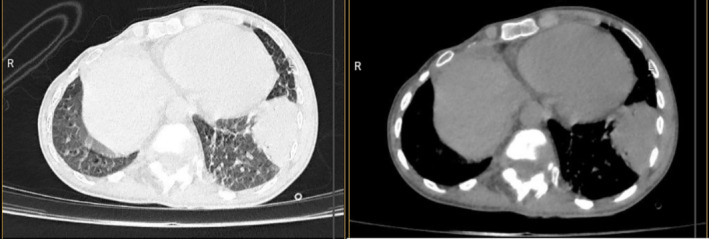
The consolidation, which is 38×58 mm in size, is in the superior part of the left lower lobe.

The new CT scan of the lungs showed an increase in the size of consolidation in the left lower lobe (38×58 mm dimensions) (Figure 2).

The patient's paranasal sinuses (PNS) CT scan did not show pathological findings, and the sputum tests were negative for Pneumocystis, Mucormycosis, and Aspergillus.

**FIGURE 3:**
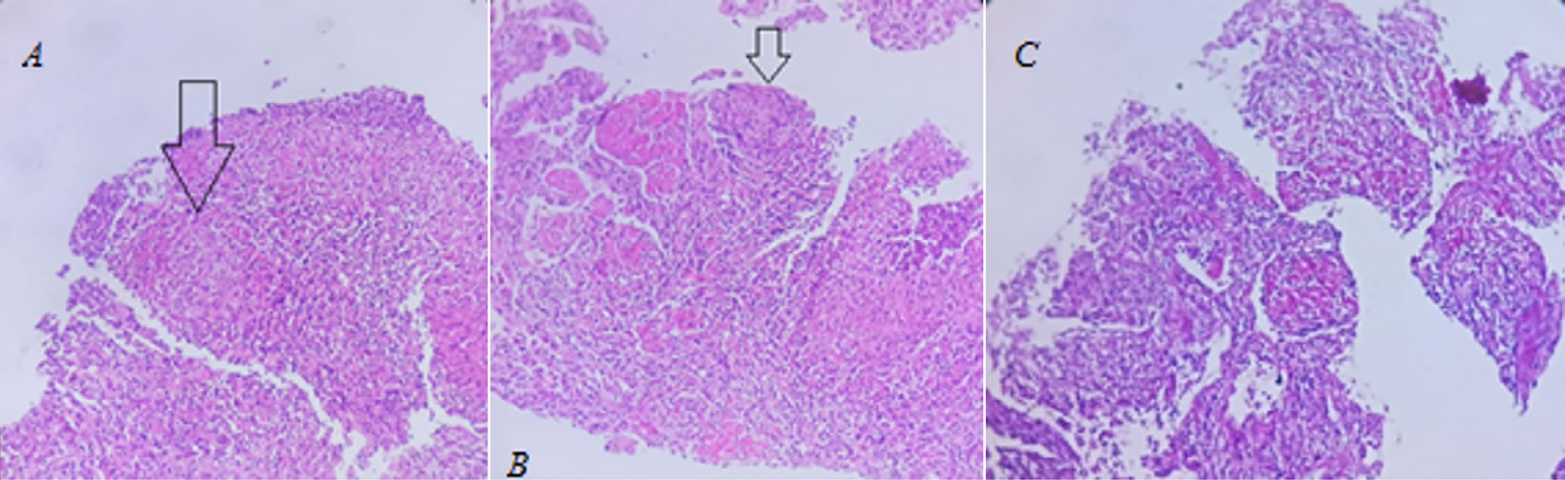
(A, B) Sections show lung tissue with granulomatous reaction without apparent caseous necrosis (arrows). (C) In PAS, staining fungal elements was not observed. The pathologic findings in transthoracic needle biopsy (TTNB) were compatible with granulomatosis infection without fungus hyphae.

Patient undergoing transthoracic needle biopsy (TTNB) under guide CT scan of left lower lobe lung consolidation. The pathology of the TTNB sample was compatible with granulomatosis infection without fungus hyphae. For identification of the cause of granulation inflammation, TB PCR on formalin‐fixed paraffin‐embedded (FFPE) block was recommended. (Figure 3).

## 4 Conclusions and Results

Twenty‐four days after the patient's admission, antibiotics were stopped due to the cessation of fever and improvement of dyspnea, and the injectable corticosteroid was changed to oral (prednisolone 25 mg daily and tapered by 5 mg/day every week until complete discontinuation). A ventilation‐perfusion (VQ) scan was performed to investigate chronic embolism, in which there was no evidence of chronic thromboembolic pulmonary hypertension (CTEPH).

A PCR of tuberculosis tissue was requested on the TTNB sample, and its result was positive. Considering the confirmation of the TB diagnosis, the patient was treated with four standard drugs (daily three tablets of fixed‐dose combination (FDC) anti‐TB therapy produced by Svizera Netherlands. Any tablet contains 150 mg of rifampin, 75 mg of isoniazid, 400 mg of pyrazinamide, and 275 mg of ethambutol), and he was referred to the tuberculosis treatment center for continued treatment after discharge.

## 5 Discussion

CGD is a primary immunodeficiency disorder caused by a defect in the gene encoding subunits of the NADPH oxidase complex, which causes defects in producing reactive oxygen species (ROS) by phagocytes [1, 6]. In this rare disease, phagocytes have defects in autophagy and activation of the inflammasome. As a result, the person becomes prone to severe infections of catalase‐positive bacteria, fungi, and granuloma tissue formation [1, 6, 7]. While based on previous studies, bacterial and fungal infections have a high prevalence in CGD patients, mycobacterial infections, including TB, have been reported less in these patients and pose significant diagnostic and therapeutic challenges [3, 8].

In the presented case, a 27‐year‐old male with known CGD developed progressive dyspnea, a productive cough, and a fever. His clinical history of recurrent Pneumonia and a previous episode of Aspergillus pneumonia aligned with the typical infectious complications seen in CGD [1]. Initial suspicions focused on bacterial or fungal etiologies; however, identifying *Mycobacterium tuberculosis* via transthoracic needle biopsy (TTNB) highlighted the necessity of considering a broader differential diagnosis in immunocompromised patients [9].

Hyperactivation of NF‐ĸB and inflammasome in CGD phagocytes leads to the production of pro‐inflammatory cytokines and inflammatory manifestations such as granuloma formation [1]. Therefore, these patients are predisposed to granulomatous lesions, which can have manifestations similar to some infections and inflammatory conditions in radiological examinations [10].

In this case, imaging studies revealed patchy consolidations and ground glass opacities, which are not specific and can be seen in both fungal infections and TB [7]. The absence of fungal elements in the histopathological examination and the positive polymerase chain reaction (PCR) for TB ultimately confirmed the diagnosis.

Although the prevalence of TB has decreased in developed countries, there are still concerns about contracting it, especially in immunocompromised people such as CGD patients, primarily since this disease can still be transmitted from endemic areas to other areas during travel or migration [11, 12]. Granuloma formation is a response of the immune system against structures that cannot be destroyed; therefore, in CGD patients, due to the defect in the formation of effective granulomas, patients are more susceptible to mycobacterial infections [13]. Among other mechanisms that can make CGD patients more susceptible to mycobacterial infections, we can mention the disruption in cytokine production and autophagy [13].

Despite broad‐spectrum antibiotic therapy, our patient had a delayed onset of fever during hospitalization, which indicates the complexity of diagnosing infections in CGD patients. Failure of initial treatment led to further evaluation and diagnosis of TB in this patient. In cases where standard treatment fails and there is clinical suspicion, tissue sampling and microbiological confirmation are necessary to establish the infection in these patients [10].

TB treatment of CGD patients requires higher precision due to their immunodeficiency and the possibility of drug interactions [14]. Standard anti‐tubercular therapy was started for our patient, which led to the clinical improvement of the patient's condition. The risk of treatment complications of TB in CGD patients may be higher due to their immunodeficiency status, and they should be closely monitored during treatment [15].

This case reveals the importance of considering TB in the differential diagnosis of CGD patients presenting with pulmonary symptoms. Interpreting clinical and radiological findings in these patients is difficult due to the underlying granulomatous inflammation, and definitive microbiological diagnoses are necessary. Early diagnosis and appropriate treatment of TB, like other infections in CGD patients, are necessary to reduce morbidity and prevent dissemination [15].

This case study provides valuable insight into the diagnosis and management of TB in immunocompromised patients like CGD. A major strength of this case study is the detailed presentation of the patient's clinical history, diagnostic workup, and management of an atypical presentation of TB in an immunocompromised patient. Also, this case underscores the importance of using advanced techniques like TTNB for definitive microbiological confirmation of atypical cases with a high suspicion of TB.

Our study was a case report; its findings cannot be generalized to all CGD patients. Also, due to pulmonary hypertension and hypoxia, we could not perform a bronchoscopy on the patient, so we had limitations in exploring concurrent opportunistic infections.

Further studies are needed to identify trends and better characterize the relationship between CGD and mycobacterial infections. In addition, investigation for well‐tailored diagnostic tools for mycobacterial infections with higher sensitivity and specificity is necessary.

## 6 Conclusion

TB diagnosis in CGD patients may create complex clinical scenarios and require more precision and careful investigation. Physicians should be aware of the atypical presentations of TB in immunocompromised patients and consider it as a potential diagnosis. This case underscores the need for comprehensive diagnostic evaluations and tailored therapeutic strategies to manage infections effectively in CGD patients.

## Author Contributions

**Davood Attaran:** conceptualization, investigation, project administration, supervision, writing – review and editing. **Shima Baniassad:** conceptualization, writing – original draft, writing – review and editing. **Zahra Behrooznia:** conceptualization, investigation, methodology, writing – original draft, writing – review and editing. **Ehsan Taheri:** conceptualization, investigation, writing – review and editing. **Soroush Attaran:** conceptualization, writing – original draft, writing – review and editing. **Amir Baniasad:** conceptualization, investigation, writing – original draft, writing – review and editing.

## Consent

Written informed consent was obtained from the patient to publish this report in accordance with the journal's patient consent policy.

## Conflicts of Interest

The authors declare no conflicts of interest.

## Data Availability

The data supporting this study's findings are available from the corresponding author upon reasonable request.
